# Supplementary figures and images for: C. elegans XMAP215/ZYG-9 and TACC/TAC-1 act at multiple times during oocyte meiotic spindle assembly and promote both spindle pole coalescence and stability
Source: PLoS Genet. 2023 Jan 6;19(1):e1010363. doi: 10.1371/journal.pgen.1010363 (PMC9851561; doi:10.1371/journal.pgen.1010363)

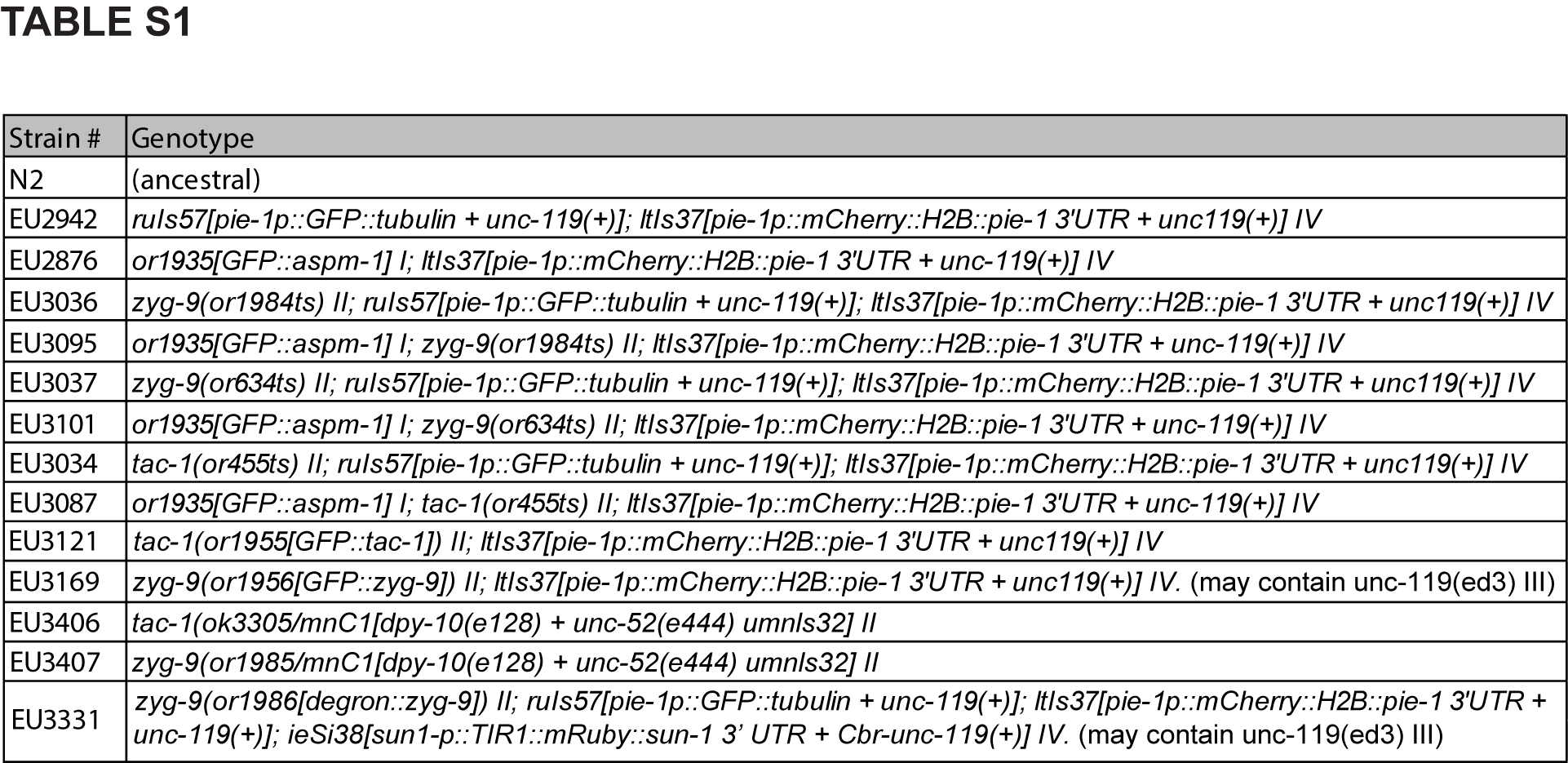

Supplement: S1 Table — (TIF) [file pgen.1010363.s001.tif]

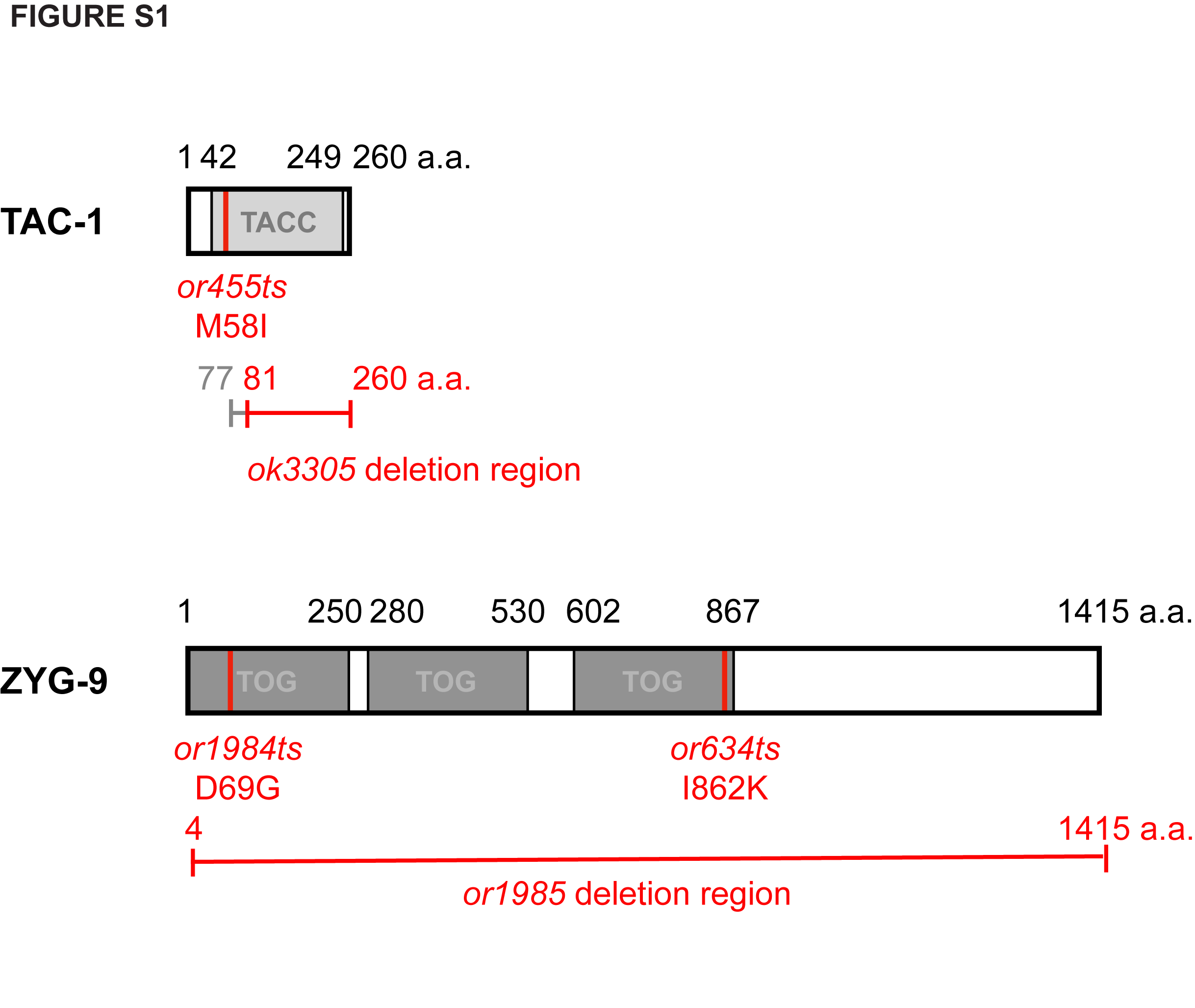

Supplement: S1 Fig — Schematic diagrams indicating locations of the mis-sense mutations in TS alleles (red vertical lines), the tac-1(ok3305) and zyg-9(or1985) deletion endpoints (horizontal red line), the TAC-1 coiled-coil TACC domain in light grey, and the ZYG-9 TOG domains in dark grey, with amino acid boundaries of domains indicated. The tac-1(ok3305) allele is a complex rearrangement with a large deletion and small 23 base pair insertion 5’ to the deletion at the junction of exon 1 and intron 1 that introduces four extra amino acids to the C-terminus of the exon 1 encoded amino acids (see WormBase). (TIF) [file pgen.1010363.s002.tif]

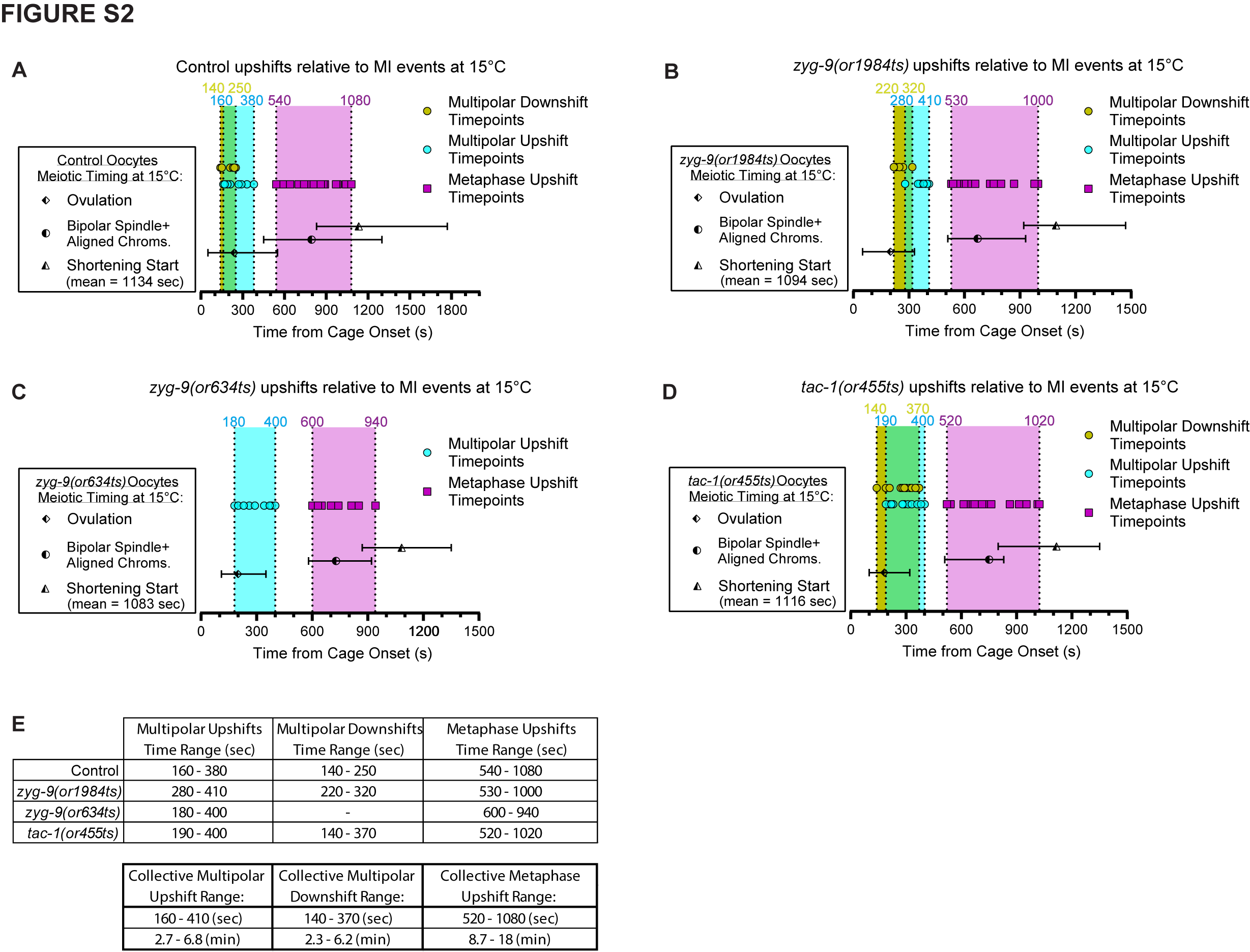

Supplement: S2 Fig — (A-D) All upshift and downshift timepoints for multipolar and metaphase temperature-shift experiments overlaid onto the timing of meiotic events at 15°C for each allele. Note that the multipolar downshifts are shifted to slightly earlier time points due to the more rapid development at 26°C prior to the downshifts. Error bars and values are mean ± the range (S1 Data). (E) Tables displaying the ranges of multipolar and metaphase temperature-shift timepoints relative to cage onset at 15°C for each allele. (TIF) [file pgen.1010363.s003.tif]

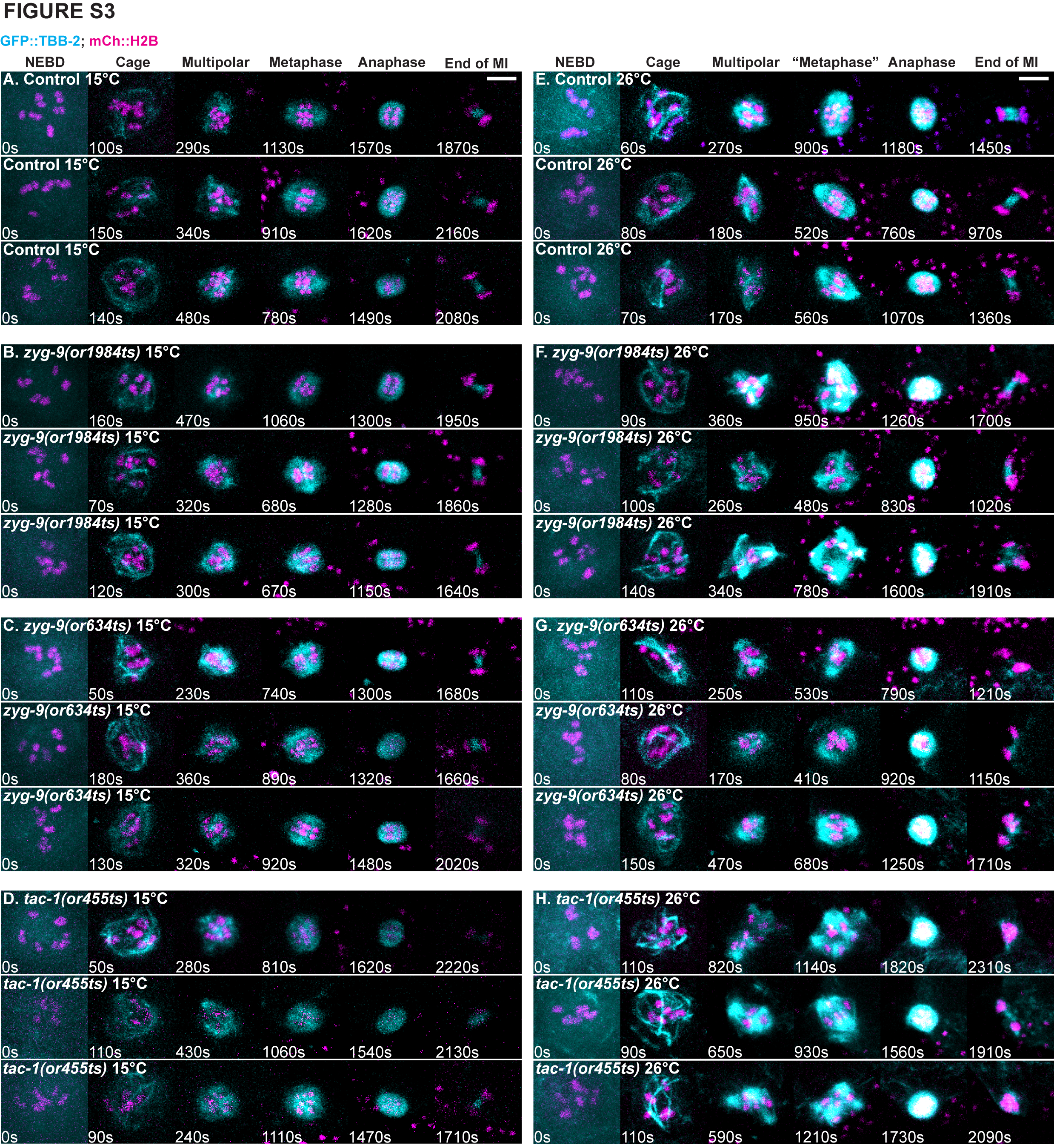

Supplement: S3 Fig — (A-H) Time-lapse maximum intensity projection images during meiosis I in live control and TS mutant oocytes expressing GFP::TBB-2 and mCherry::H2B to mark microtubules and chromosomes, at 15°C (A-D) and at 26°C (E-H). In this and in all subsequent meiosis I time-lapse image series, t = 0 is labeled NEBD and is the timepoint immediately preceding the appearance of microtubule bundles forming the cage structure. See the Materials and Methods for a description of the assembly stages and frame selections in this and subsequent supplemental figures. Scale bars = 5 μm. (TIF) [file pgen.1010363.s004.tif]

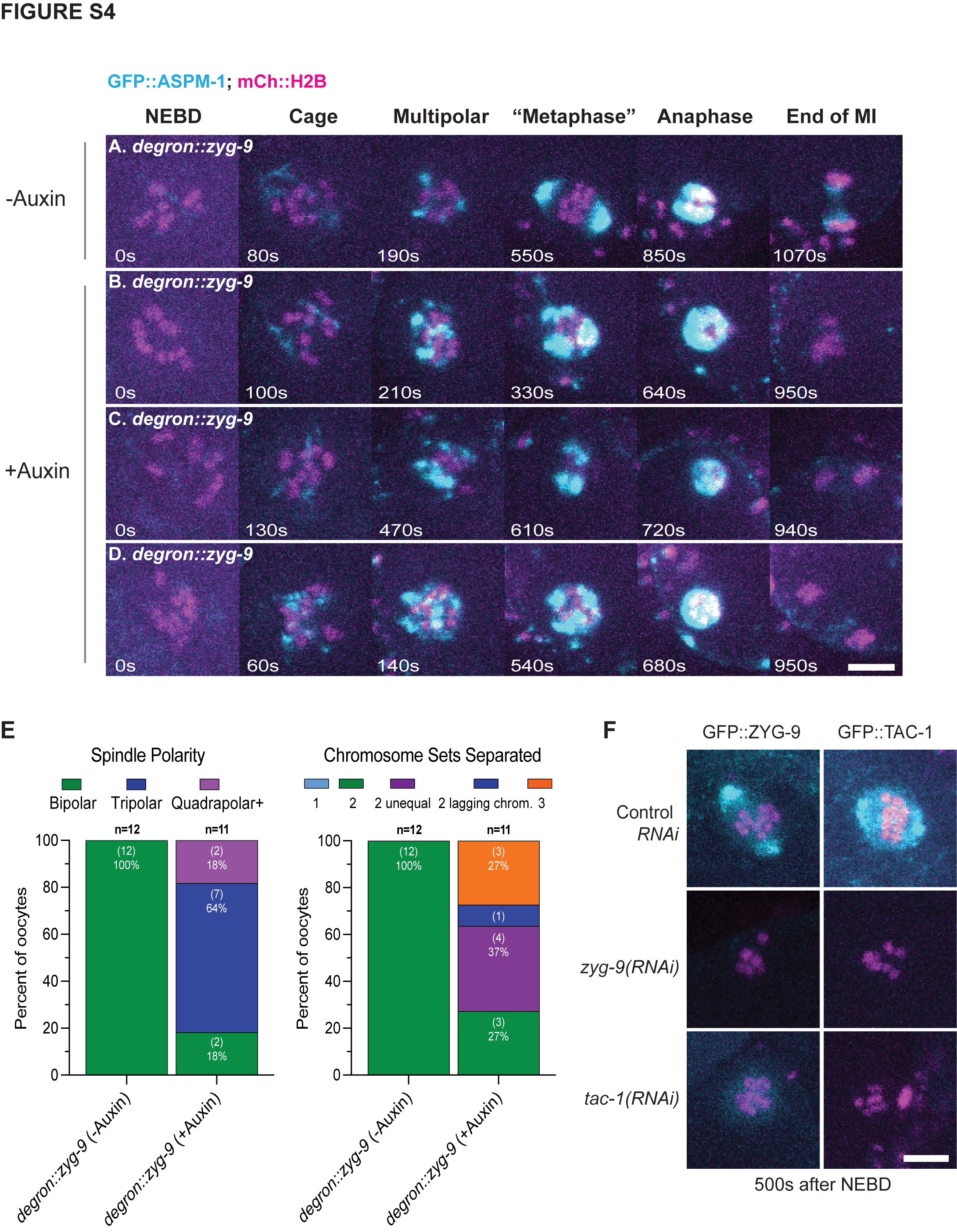

Supplement: S4 Fig — (A-D) Time-lapse maximum intensity projection images of live control (-Auxin) and auxin-induced knockdown (+Auxin) of degron-tagged endogenous zyg-9 locus at indicated stages. mCherry::H2b in magenta; GFP::ASPM-1 in teal. (E) Bar graphs quantifying the spindle polarity and chromosome separation defects in control and auxin treated oocytes. (F) RNAi knockdowns using previously reported conditions and endogenous GFP fusion strains [21]. As also reported by others [44], all GFP::TAC-1 signal is lost from spindle microtubules and poles after zyg-9 RNAi, while GFP::ZYG-9 is lost from poles but not spindle microtubules after tac-1(RNAi). Scale bars = 5 μm. (TIF) [file pgen.1010363.s005.tif]

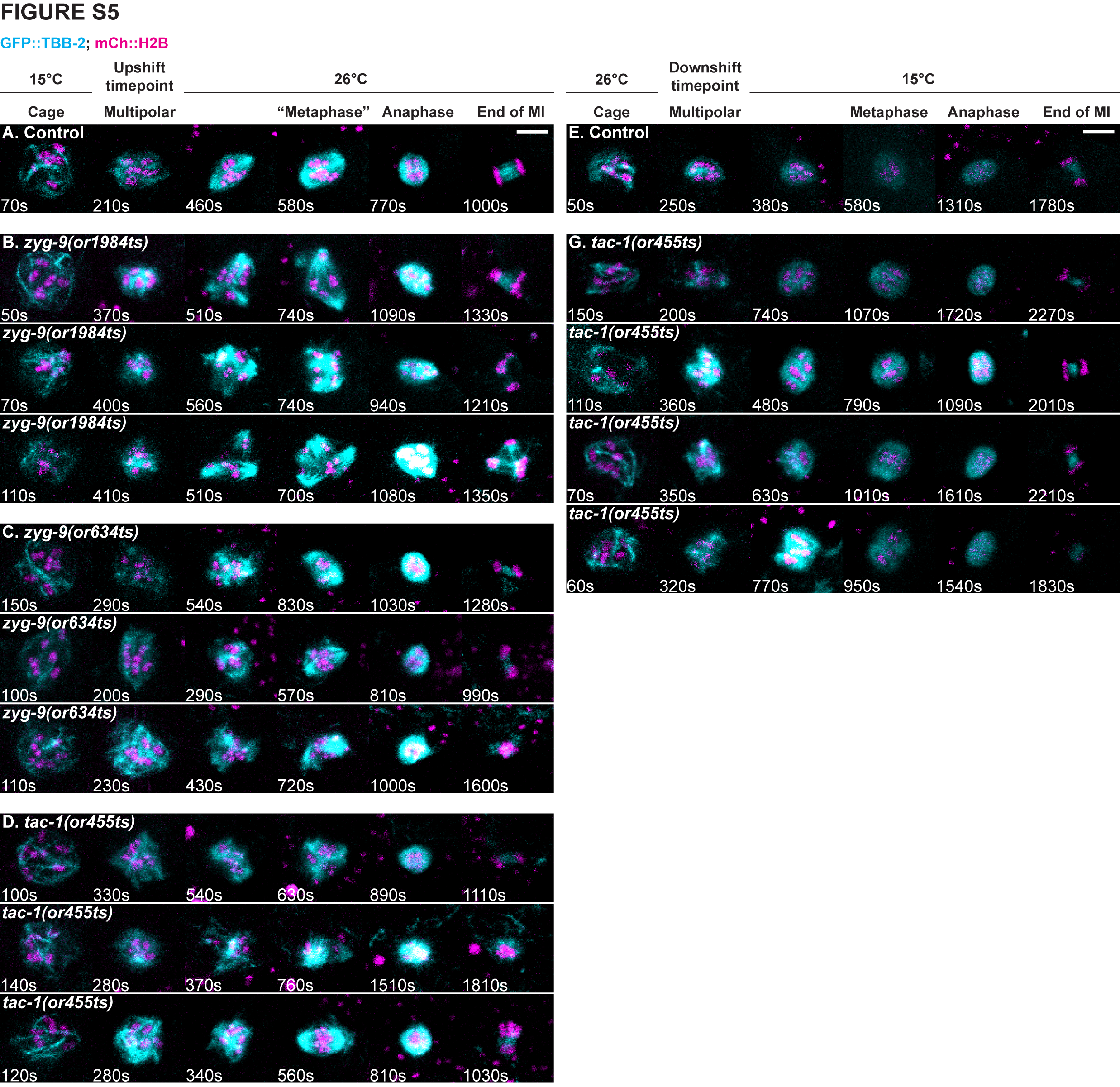

Supplement: S5 Fig — (A-G) Time-lapse maximum intensity projection images of live control and TS mutant oocytes upshifted to 26°C (A-D) or downshifted to 15°C (E-G) during the multipolar stage, in oocytes expressing GFP::TBB-2 and mCherry::H2B. Oocytes depicted in G have cage structure defects (for an example, see S9 Movie). Scale bars = 5 μm. (TIF) [file pgen.1010363.s006.tif]

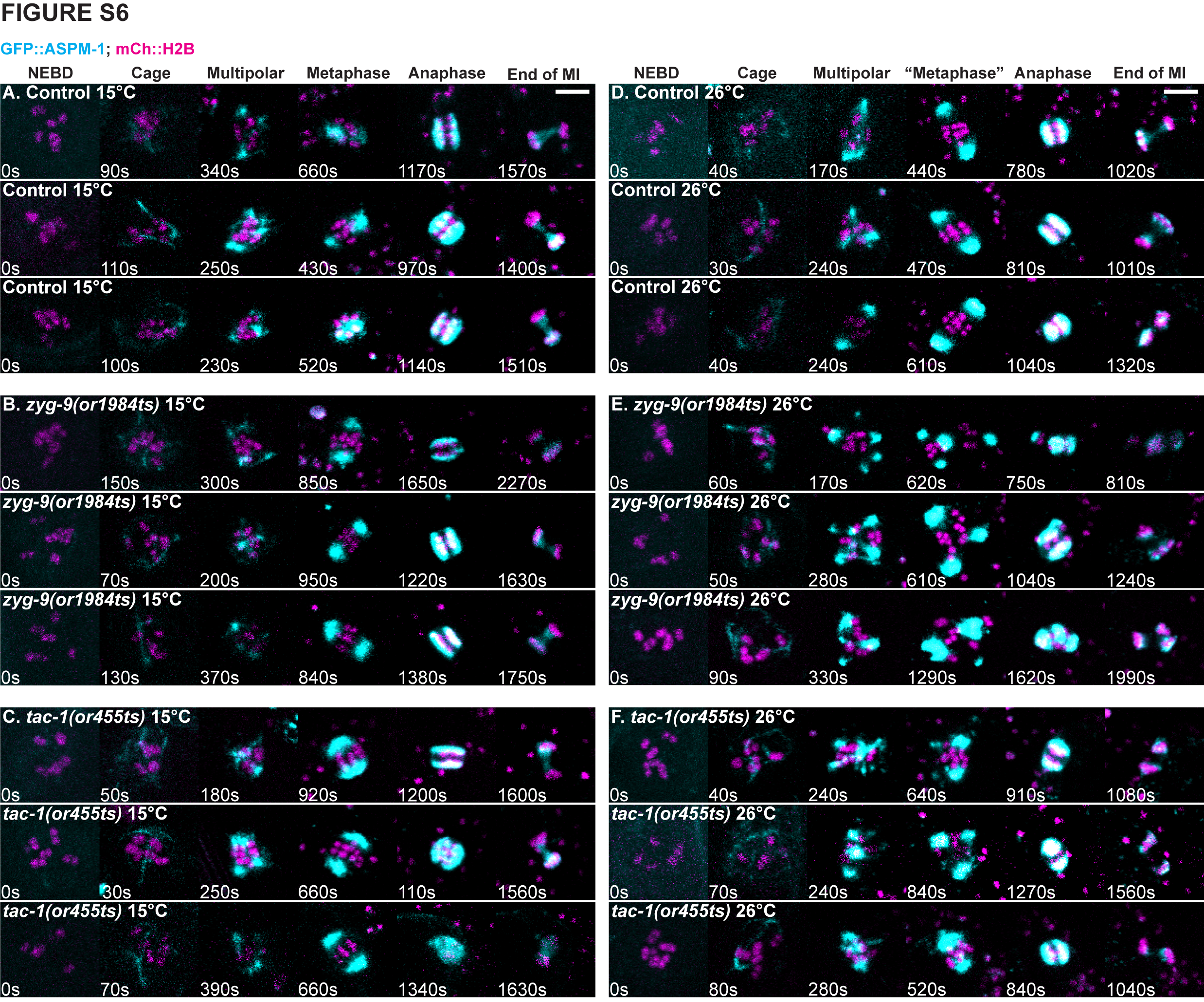

Supplement: S6 Fig — (A-F) Time-lapse maximum intensity projection images of live control and TS mutant oocytes expressing GFP::ASPM-1 and mCherry::H2B to mark spindle poles and chromosomes, at 15°C (A-C), at 26°C (D-F). Scale bars = 5 μm. (TIF) [file pgen.1010363.s007.tif]

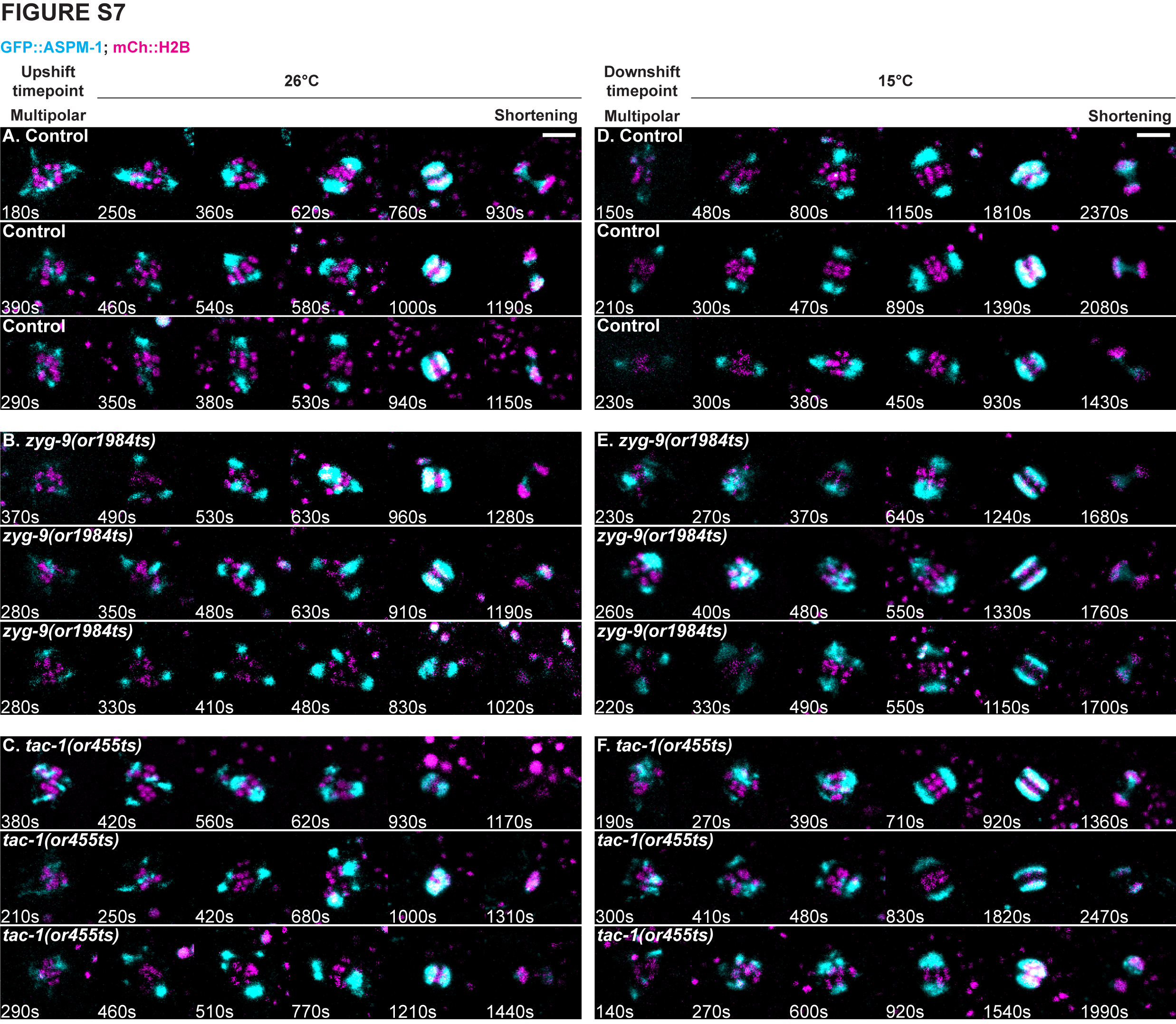

Supplement: S7 Fig — (A-F) Time-lapse maximum intensity projection images of live control and TS mutant oocytes expressing GFP::ASPM-1 and mCherry::H2B to mark spindle poles and chromosomes, and upshifted to 26°C (A-C) or downshifted to 15°C (D-F) during the multipolar stage. Montage frames highlight pole coalescence dynamics during the multipolar stage through to the onset of spindle shortening. Scale bars = 5 μm. (TIF) [file pgen.1010363.s008.tif]

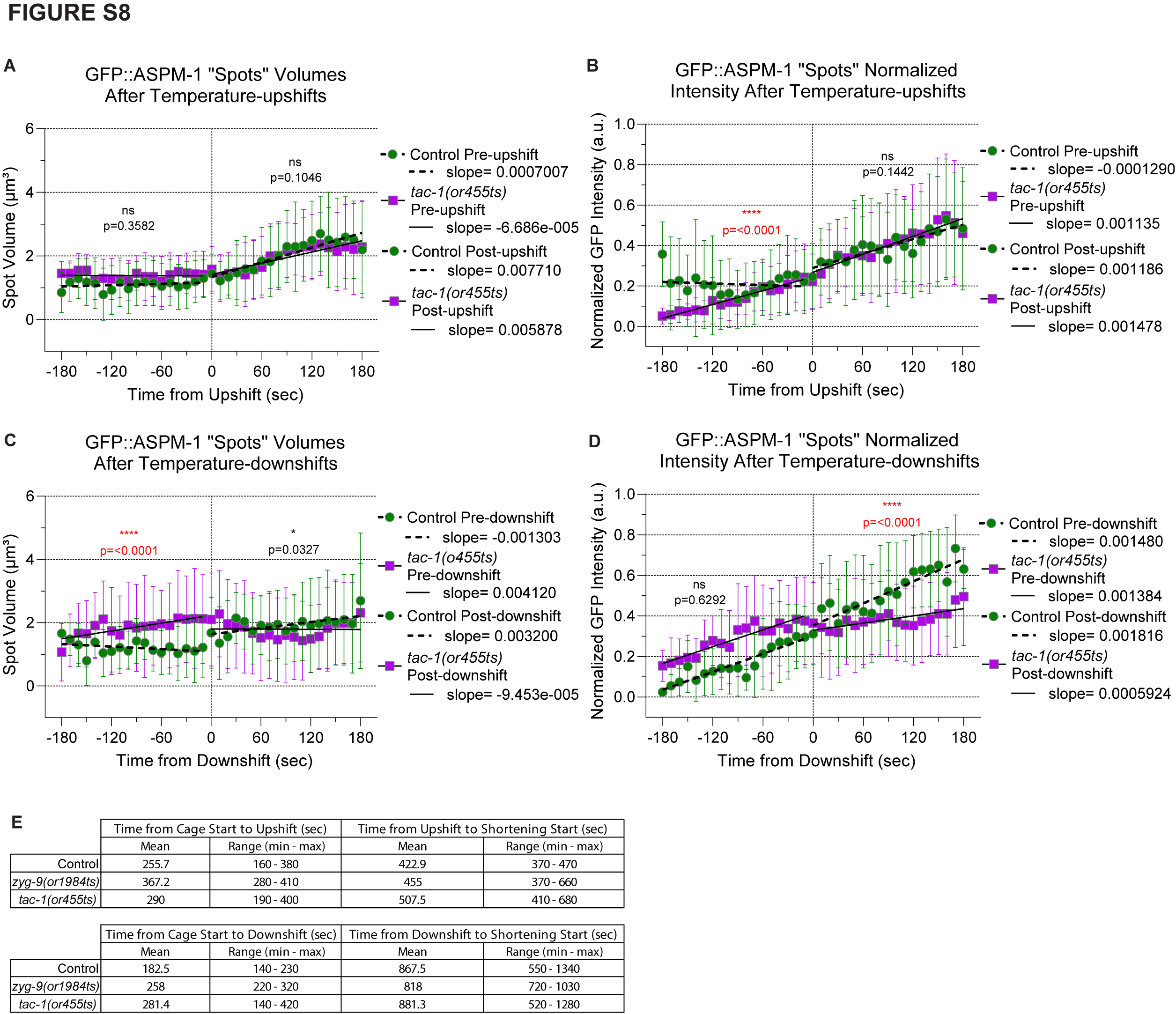

Supplement: S8 Fig — (M-P) Quantification of control and tac-1(or455ts) GFP::ASPM-1 foci volume and intensity (see Materials and Methods) pre- and post-multipolar upshift (A, B) and downshift (C,D). Slopes were compared using a two-tailed t-test to calculate P-values (S4 Data). (E) Table showing the time elapsed pre- and post-multipolar upshift (upper table) and multipolar downshifts (lower table) for control and TS mutants. *, P <0.05; ****, P <0.0001. (TIF) [file pgen.1010363.s009.tif]

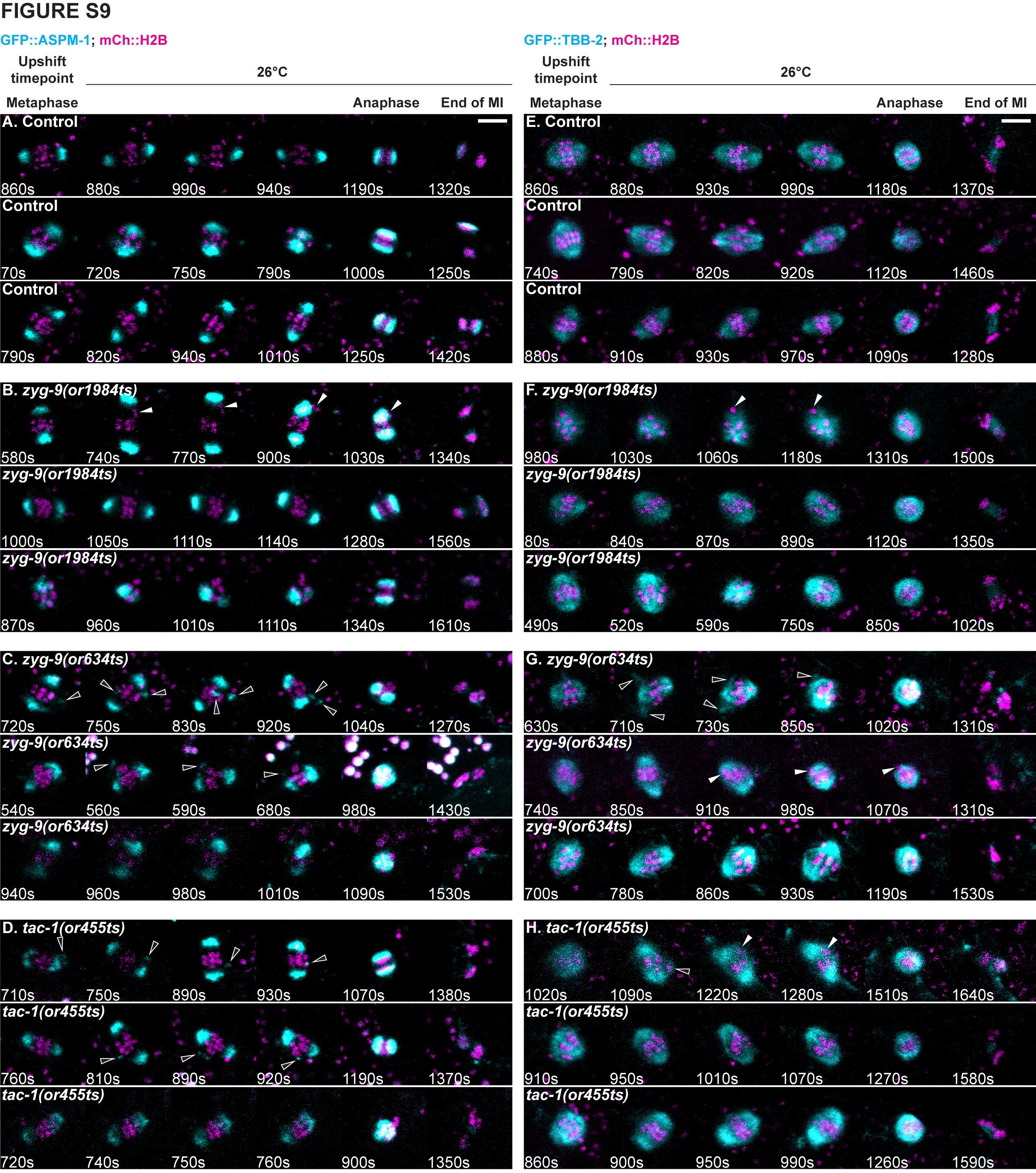

Supplement: S9 Fig — (A-H) Time-lapse maximum intensity projection images of live control and TS mutant oocytes upshifted at metaphase and expressing either GFP::ASPM-1 and mCherry::H2B (A-D) or GFP::TBB-2 and mCherry::H2B (E-H). Montage frames highlight defects following metaphase upshift through to the end of meiosis I. White outlined arrowheads denote ectopic spindle poles and solid white arrowheads indicate chromosome congression errors. The montage in B (top row) is also depicted in Fig 7B. Scale bars = 5 μm. (TIF) [file pgen.1010363.s010.tif]

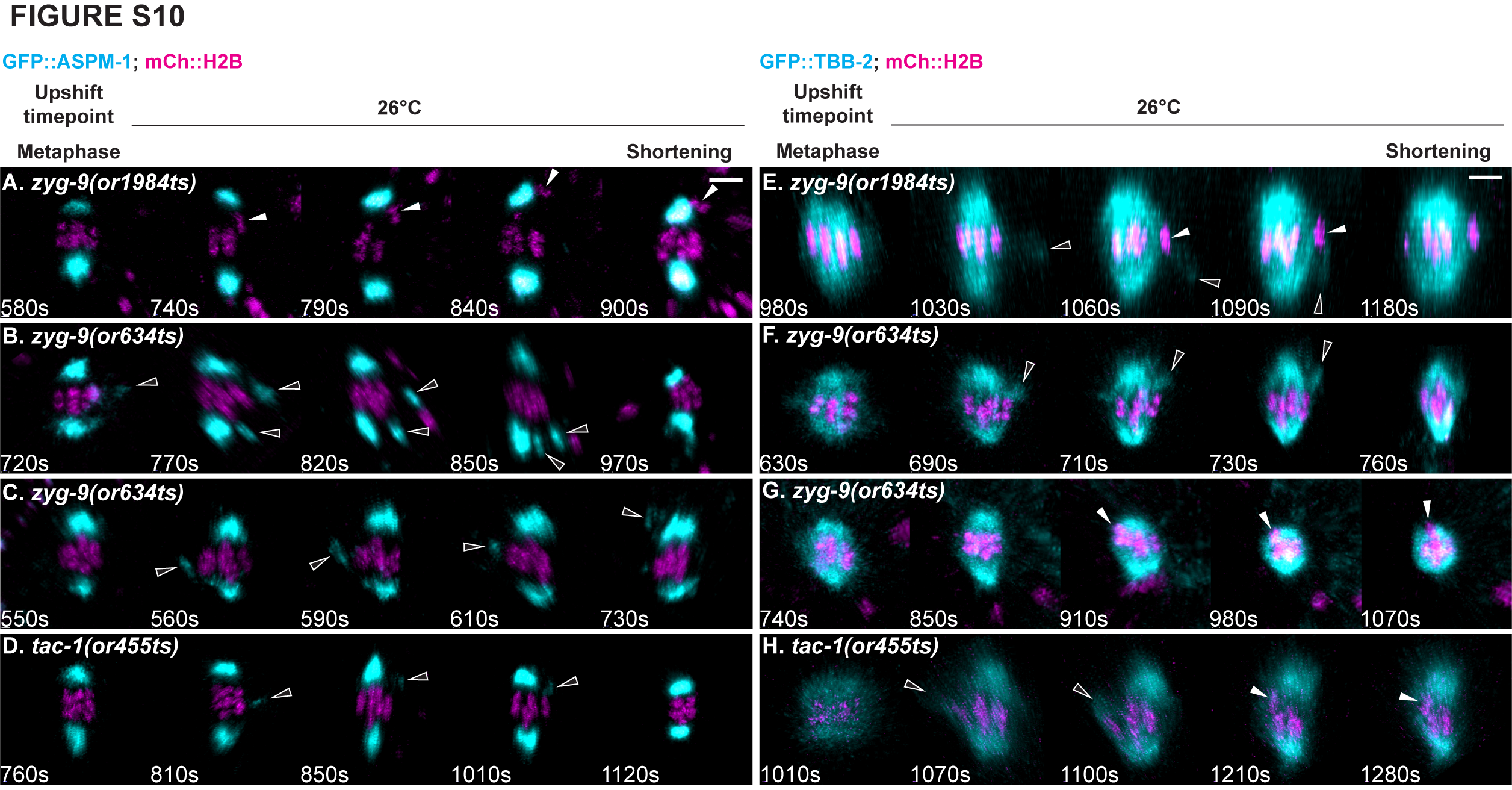

Supplement: S10 Fig — (A-H) Imaris rotated and snapshot projected time-lapse images (see Materials and Methods) of live TS mutant oocytes upshifted at metaphase expressing GFP::ASPM-1 and mCherry::H2B (A-D) or GFP::TBB-2 and mCherry::H2B (E-H). Montage frames highlight defects following metaphase upshift through to spindle shortening. White outlined arrowheads denote ectopic spindle poles; solid white arrowheads indicate chromosome congression errors. Imaris montages of A-H are of the same oocytes shown in the top rows of S7 Fig as maximum intensity projection montages B-H. Scale bars = 5 μm. (TIF) [file pgen.1010363.s011.tif]

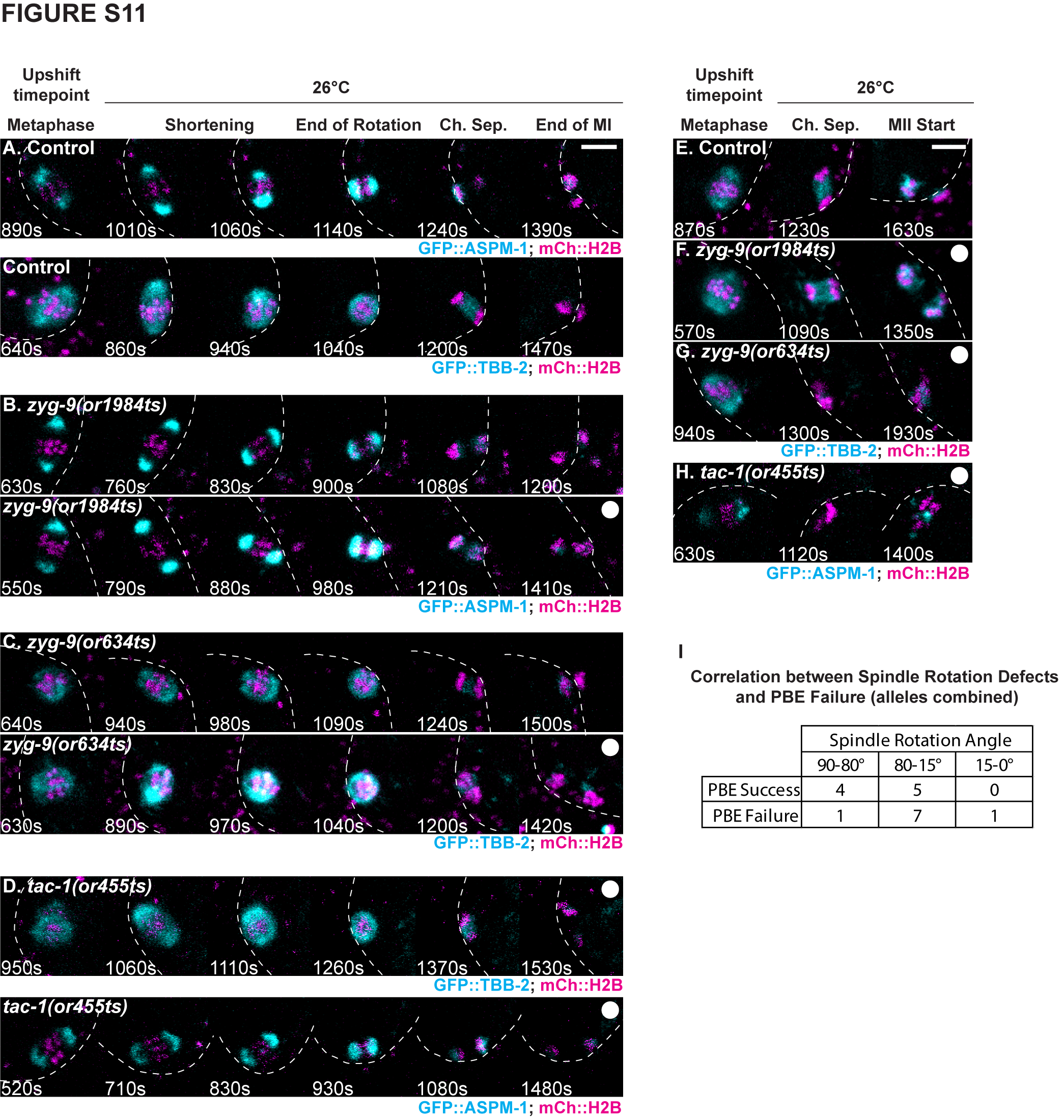

Supplement: S11 Fig — (A-H) Time-lapse maximum intensity projection images of live control and TS mutant oocytes expressing GFP::ASPM-1 and mCherry::H2B or GFP::TBB-2 and mCherry::H2B upshifted at meiosis I metaphase. Dashed lines depict the oocyte cortex. Montages with a white circle in the last frame indicate failed polar body extrusion. The montage in B (top row) is also depicted in Fig 5B. Montages in E-H are also depicted in S7E Fig bottom row, Fig 5I, S7G Fig middle row, and S7D Fig bottom row, respectively. (I) Table showing the correlation between spindle rotation defects and polar body extrusion failure. Only oocytes in which both spindle rotation and polar body extrusion could be scored are included. Scale bars = 5 μm. (TIF) [file pgen.1010363.s012.tif]

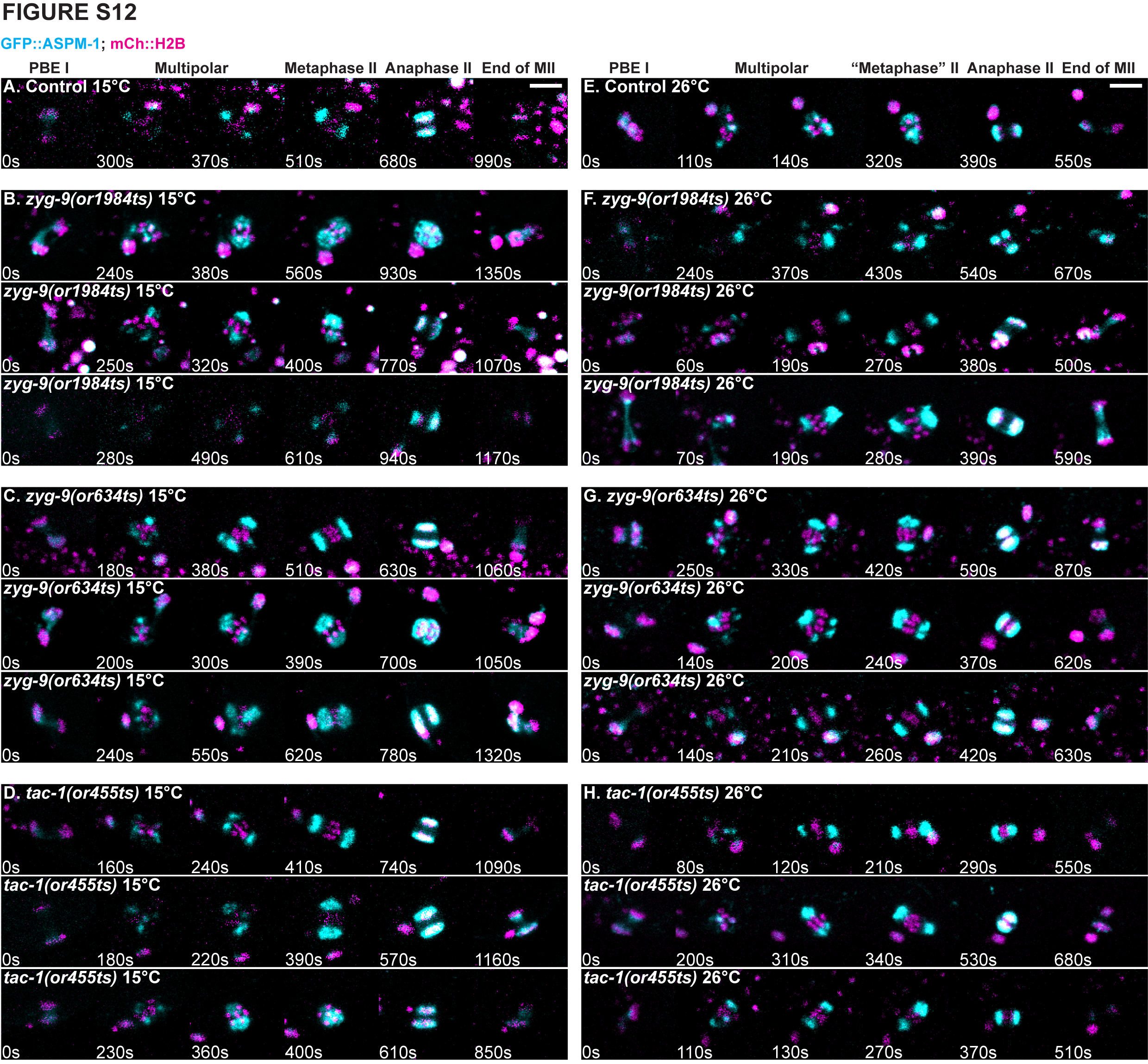

Supplement: S12 Fig — (A-H) Time-lapse maximum intensity projection images during meiosis II of live control and TS mutant oocytes expressing GFP::ASPM-1 and mCherry::H2B at 15°C (A-D) and at 26°C (E-H). t = 0 is the timepoint when meiosis I chromosome separation ends. Scale bars = 5 μm. (TIF) [file pgen.1010363.s013.tif]

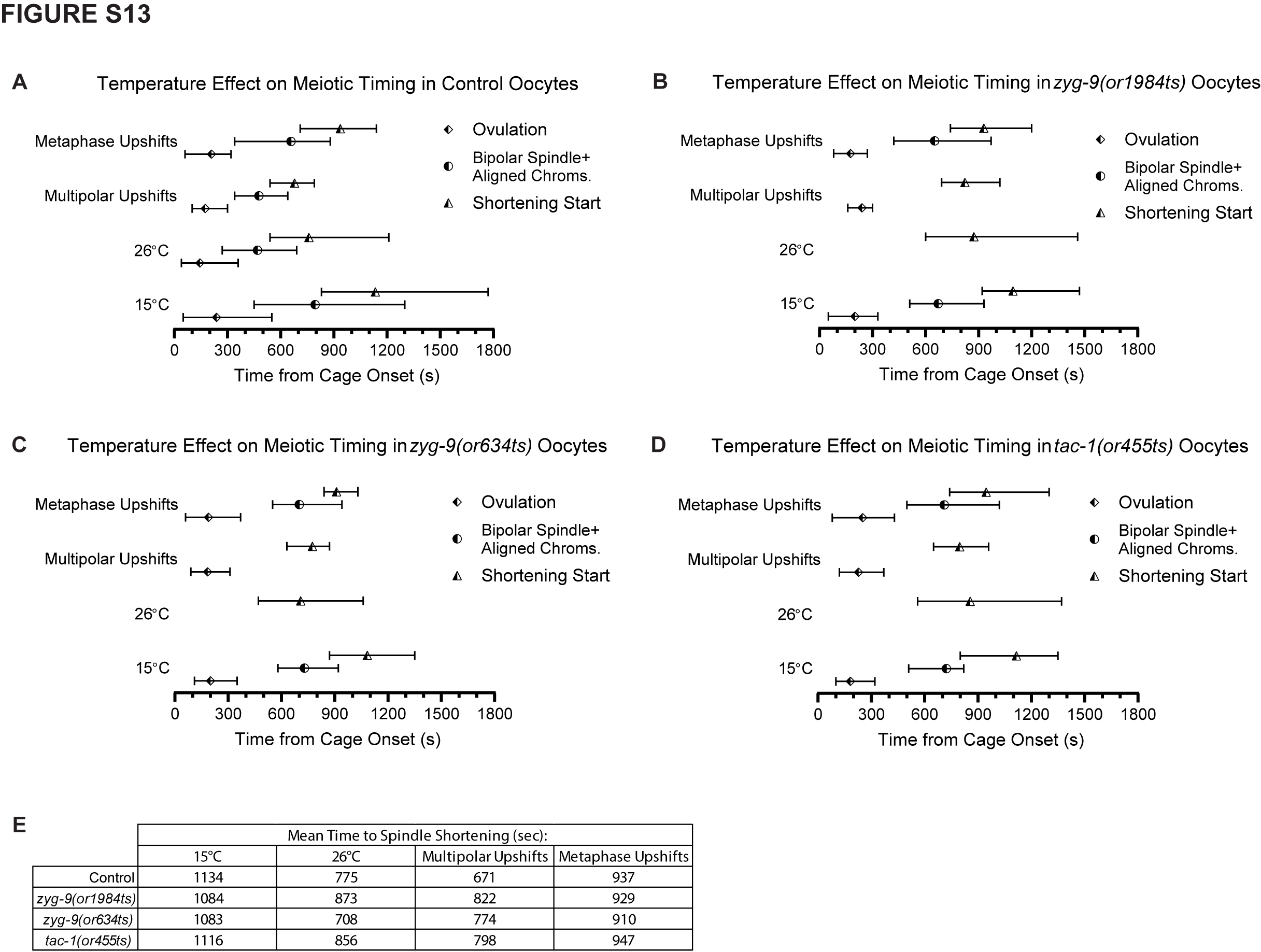

Supplement: S13 Fig — (A-D) The timing of meiotic events in oocytes maintained at 15°C or 26°C, and in oocytes that underwent multipolar and metaphase upshifts, in control and TS mutant oocytes. Error bars and values are mean ± the range. (E) Table showing the mean time to spindle shortening in control and TS mutant oocytes for each temperature condition (S1 Data). Spindle bipolarity and chromosome alignment is rarely achieved in multipolar upshifted or TS mutant oocytes at 26°C and so is not scored; ovulation does occur in TS mutants at 26°C but was not scored. (TIF) [file pgen.1010363.s014.tif]
