# Supplementary material for: C. elegans XMAP215/ZYG-9 and TACC/TAC-1 act at multiple times during oocyte meiotic spindle assembly and promote both spindle pole coalescence and stability
Source: PLoS Genet. 2023 Jan 6;19(1):e1010363. doi: 10.1371/journal.pgen.1010363 (PMC9851561; doi:10.1371/journal.pgen.1010363)
Supplement: Attachment — Submitted filename: Response to Reviewers PLOS Genetics Revised.docx [file pgen.1010363.s044.docx]

**Response to All Reviewer Comments**

Reviewers’ comments in blue; responses in black; *revised text excerpts in italics*

We thank both reviewers for their careful reading of our manuscript and for their thoughtful and very helpful comments. We have made revisions that address most of the suggestions and have modified the text to clarify our interpretations as follows.

**Reviewer 1 comments.** Previous work from the same lab has shown that ZYG-9 (homolog of a conserved microtubule-binding protein that has been studied extensively in many species) is required for C. elegans oocyte meiotic spindle poles to coalesce into exactly two poles. Having exactly two poles is important for accurate reduction of chromosome number during meiosis. Therefore this is a very significant problem that is likely relevant in other species. This manuscript includes very careful analysis of live imaging data. The work is mostly very rigorous, adds reproducibility to previous work, and adds many details to the zyg-9 phenotype. However, the work does not shed significant light on the mechanism of pole coalescence and does not really add to our understanding of what a pole is. ASPM from other species has been shown to bind microtubule minus ends but EM tomography has shown that minus ends are distributed equally throughout these spindles. Three major strengths of the manuscript are 1) it is very well written and mostly does a good job of citing relevant previous work; 2) the use of rapid temperature upshift and downshift on ts mutants to separate early and late direct functions and 3) the finding that the rate of growth of ASPM foci is faster in upshifted zyg-9 mutants.

We agree these are key strengths of our manuscript. We also think that documenting that ZYG-9/TAC-1 likely act during the process of pole coalescence itself, and that they also are required for pole stability and hence the maintenance of chromosome congression during metaphase, are important contributions. We also want to emphasize the defects in spindle rotation and polar body extrusion in *zyg-9* and *tac-1* mutants have not been reported previously and are worthy of further investigation, as we point out in the Discussion. Moreover, our new data describing for the first time null alleles of both loci is another important contribution. While our observation that pole foci growth dynamics are changed but only in *zyg-9(or1984ts)* might not be relevant to the other spindle assembly defects (see comments below), we do think they are interesting with respect to the phase transition properties observed during mammalian oocyte meiotic spindle assembly and therefore potentially of value beyond any role in spindle assembly. As detailed below, we appreciate the input on citing relevant literature and now include reference to publications that we failed to cite in our original submission.

Unfortunately, there are minor weaknesses associated with two of these strengths. Unlike RNAi or degron depletions, there is no way to directly assess the extent of inactivation in a temperature shift of a ts mutant because the protein might still be present but be partially denatured. For some experiments detailed below, the conclusions are not justified because shorter temperature shifts might cause less inactivation. The increased rate of ASPM focus growth observed in zyg-9(ts) was not observed in tac-1(ts). Because the pole instability and chromosome segregation defects were observed in both zyg-9(ts) and tac-1(ts), the increased rate of focus growth cannot be the cause of the larger scale, significant phenotypes. Overall, the work is very rigorous but provides only limited new insight into how poles coalesce or how zyg-9/tac-1 function.

We thank the reviewer for pointing out our flawed logic on this point in our original submission. We now acknowledge this caveat but also discuss how this change in pole dynamics might nevertheless be related to the spindle pole coalescence and stability defects caused by loss of ZYG-9 or TAC-1. We have revised the relevant text in the Results section as follows (new text *underlined*):

*While we did not detect these differences in tac-1(or455ts) mutant oocytes (Fig. S8, Movie S15), the rapid growth of small GFP::ASPM-1 foci into more prominent foci after the temperature upshifts in zyg-9(or1984ts) mutant oocytes raises the possibility that ZYG-9 and TAC-1 may promote pole coalescence by limiting the growth of pole foci, in addition to preventing their splitting (see Discussion).*

And in the Discussion:

*It is important to note that we observed these effects on pole growth dynamics only after temperature shifts in zyg-9(or1984ts) and not in tac-1(or455ts) oocytes, while we observed the splitting of pole foci, failures to establish spindle bipolarity, and failures to properly separate chromosomes after both zyg-9(or1984ts) and tac-1(or455ts) upshifts. Our failure to detect such changes in ASPM-1 pole foci dynamics in tac-1(or455ts) may indicate that such changes in foci dynamics are not required for the spindle and chromosome separation defects that zyg-9 and tac-1 TS mutants both share. However, the spindle bipolarity and chromosome separation defects are more highly penetrant in zyg-9(or1984ts) than in tac-1(or455ts) oocytes, and it is possible that more complete elimination of TAC-1 might reveal similar detectable changes in pole foci dynamics and more penetrant spindle defects. Moreover, it also is possible that the mutation in TAC-1, while not affecting pole foci growth dynamics, may disrupt another function that also is required for spindle assembly and function. Indeed, the higher penetrance of the spindle polarity and chromosome separation defects in zyg-9(or1984ts) oocytes, and the higher penetrance of the cage defect in tac-1(or455ts) oocytes, suggest that these mutations differently alter complex function. Alternatively, the change in pole foci growth dynamics might reflect properties of ZYG-9 that are not related to the spindle assembly and chromosome separation defects we and others have documented in zyg-9 mutant oocytes, and it is further possible that tightly linked second site mutations outside of the zyg-9 locus are responsible for the altered dynamics in pole growth. We speculate that these conditional and partial loss-of-function alleles differently alter complex function, and that the failure to detect the changes in pole foci growth properties associated with the weaker partial loss-of-function tac-1 mutation does not preclude limits to pole foci growth being important for pole coalescence and stability.*

Introduction
Because the introduction states that the significance of this work is showing that zyg-9/tac-1 is required to maintain spindle structure after assembly, it would be appropriate to cite Cavin-Meza 2022 who showed with a dhc-1 degron that dynein is required after spindle assembly to maintain spindle structure.

We thank the reviewer for noting this omission on our part and now include reference to this work as follows (new text *underlined*):

*The numerous defects observed upon loss of ZYG-9 or TAC-1 suggest that this complex may have multiple separate requirements during oocyte meiotic cell division. However, the use of non-conditional mutations or knockdown methods to assess gene requirements precludes determining whether each defect represents a separate requirement, or if instead the later defects are indirect consequences of earlier ones. The use of CRISPR/Cas9 genome editing to degron-tag genes for auxin-inducible degradation now makes it possible to engineer conditional loss of function alleles throughout the genome (Zhang et al., 2015). Indeed, a recent study has shown that degron-tagged dynein can be rapidly degraded and knocked down in function with only a few minutes of auxin treatment, with knockdown at metaphase during C. elegans oocyte meiosis I cell division, documenting a late role for dynein in the maintaince of spindle structure (Cavin-Meza et al., 2022).*

It would be appropriate to cite an example where something new was discovered with a fast acting ts mutant in C. elegans. Using metaphase-arrested embryos, Wolf et al. 2022 showed that klp-18 is required to maintain bipolarity after assembly and McNally et al. 2014 showed that MEI-1 is required to maintain chromosome alignment after congression. It would enhance the readers understanding of the significance of the current work if a paper was cited in which continuous function was NOT required and it was shown that the negative result was not due to incomplete depletion/inactivation

We thank the reviewer for pointing out these omissions. While we have not cited a paper showing that a gene function is definitively required only early for a late defect, our prometaphase temperature up and down shifts do strongly argue that the later spindle polarity defects are independent of the early cage defects. While there is not a perfect correlation between cage structure and spindle polarity defects, which suggests they may occur independently, our temperature shifts provide more compelling support for their independence. One cannot easily conclude that any TS mutation, or any other manipulation to reduce gene function, results in a null phenotype at the restrictive temperature or condition; we are not aware of any publications that document a conditional null condition that could provide an example of a definitive early-only requirement for a later defect. Rather, our goal here has been to demonstrate that some later defects are independent of earlier defects. We also clearly state that we do not think that our TS alleles completely eliminate gene function, even after longer times at the restrictive temperature. Finally, we do now include, as suggested, citations to both Wolf et al 2022 and McNally et al 2014 as follows (new text *underlined*):

*Furthermore, some fast-acting TS alleles also are reversible, with protein function rapidly restored upon shifting from restrictive to permissive temperatures, adding to their usefulness (Severson et al., 2000). In C. elegans oocytes, temperature upshift experiments with TS alleles have been used to document a role for MEI-1/katanin the maintenance of chromosome congression (McNally et al 2014), and for the kinesin X family member KLP-18 in the maintenance of spindle bipolarity (Wolf et al., 2022). To better define the requirements for ZYG-9 and TAC-1 during oocyte meiotic cell division, we have taken advantage of fast-acting TS alleles, with live imaging and fluorescent protein fusions, to identify multiple and separate stage-specific meiotic requirements for these two widely conserved regulators of microtubule dynamics.*

We also have revised our Discussion to more accurately reflect the relative virtues of degron tagging and fast-acting TS alleles, as follows (new text *underlined*):

*The recent development of CRISPR-mediated degron tagging of endogenous loci for temporally controlled, auxin-mediated protein degradation now provides a powerful tool for engineering conditional loss of function alleles throughout the C. elegans genome* *(Zhang et al., 2015; Dickinson and Goldstein, 2016). Degron tagging also provides impressive temporal resolution, with tagged proteins substantially knocked down after only 20 to 30 minutes of exposure to auxin (Zhang et al., 2015; Divekar et al., 2021), and in isolated early C. elegans zygotes after only a few minutes (Wignall 2022 eLife). Fast-acting TS alleles do provide some advantages. In some cases, they may reduce gene function even more rapidly, within seconds, and they can often be used for complementary temperature-upshift/downshift experiments to define temperature-sensitive periods. Moreover, with TS alleles one can more readily modulate the extent to which gene function is reduced by growth at intermediate temperatures, for example to sensitize backgrounds for modifier screens (O’Rourke et al. 2007), while the utility of degron tagging is sometimes limited by the tag promote some degradation and reduction of gene function even in the absence of auxin treatment* (Schiksnis et al., 2020)*. Nevertheless, degron tagging now makes conditional gene knockdown much more widely available for identifying C. elegans gene requirements. With further advances in the increasingly powerful ability to predict protein structure based on primary amino acid sequence, perhaps it will become feasible to accurately predict missense mutations that are likely to generate fast-acting, temperature-sensitive mutations, further adding to our ability to temporally manipulate gene function in model organisms.*

Results

“These results indicate that any further defects observed after temperature upshifts are due to inactivation of the mutant protein.” This is not the correct conclusion from this result. Complementation of the phenotype with a wt transgene would support this stated conclusion. The conclusion might be that “any further defects observed after temperature upshifts correspond to the observed embryonic lethality”.

We agree that complementation with a WT transgene is the most conclusive approach to verify causality here, but we have instead chosen to isolate and characterize deletion alleles that appear to be null for both loci, we now fully document the recessive nature of these null and our TS alleles, and we show that our TS alleles fail to complement the deletion alleles. We also now summarize what was reported previously with respect to the identification of these mutant alleles in Bellanger et al JCS 2007, which was admittedly minimal. We believe these alternative approaches suffice to verify that the mis-sense mutations we have identified in *tac-1* and *zyg-9* are responsible for the mutant phenotypes we describe.

We in fact do not know which mutant phenotypes are responsible for the embryonic lethality and have not tried to address that issue by monitoring embryonic viability after different upshifts and downshifts.

We also now include analysis of a degron-tagged allele of *zyg-9* to further verify that the spindle defects we describe are due to reduced *zyg-9* function.

We also have shown that the RNAi conditions we used in a previous publication to document similar requirements results in a substantial reduction in the expression levels of GFP fusions to the endogenous *zyg-9* and *tac-1* loci.

We have added new text in two places within the first section of the Results to describe these new data (new text *underlined)*:

*The zyg-9(or634ts) and tac-1(or455ts) alleles were previously reported to genetically map to the center and right arms of chromosome II, respectively, where the corresponding loci are located, and zyg-9(or634ts) failed to complement the previously isolated allele zyg-9(b244) (Bellanger et al., 2007). To further verify that the identified missense mutations in zyg-9 and tac-1 are responsible for the phenotypes we have observed at the restrictive temperature, we obtained the previously isolated and likely null allele tac-1(ok3305), and we used CRISPR/Cas9 to generate a zyg-9 null allele, or1985, that removes nearly the entire zyg-9 open reading frame (Figure S1; see Materials and Methods). Brood analyses indicated that complete loss of zyg-9 or tac-1 function results in a recessive, maternal-effect, embryonic-lethal phenotype, although there may be some haploinsufficiency at 26˚C (Table 1). In genetic crosses the zyg-9 and tac-1 TS alleles failed to complement their respective deletion alleles at the restrictive but not at the permissive temperature (Table 1; see Materials and Methods).*

*To further verify that the defects we observed in the zyg-9 TS mutant oocytes at the restrictive temperature are caused by loss of ZYG-9 function, we used CRISPR/Cas9 to degron tag the endogenous zyg-9 locus (Figure S1; see Materials and Methods). After auxin treatment, we observed oocyte meiotic spindle assembly defects that also resembled those reported after RNAi knockdowns (Fig. S4), and that we have observed in TS mutant oocytes at the restrictive temperature (Figs. 1, S3, Movie S2). We also examined the expression levels of GFP fusions to ZYG-9 and TAC-1 after using the same feeding RNAi knockdown conditions as we used previously to identify requirements for these two genes (Chuang et al 2020), and we observed a substantial reduction in GFP signal (Fig. S4), further confirming that the phenotypes observed are due to loss of gene function. While these different knockdown methods likely do not entirely eliminate zyg-9 and tac-1 function, they all clearly document similar requirements for the encoded proteins during oocyte meiotic spindle assembly.*

Finally, we provide extensive new data on the TS and the new *zyg-9* and *tac-1* deletion alleles both in Table 1 and also in the Methods as follows (new text *underlined*):

*The zyg-9(or1984ts) allele was isolated in a chemical mutagenesis screen (Encalada et al., 2000; O’Rourke et al., 2011), but has not been published previously. The zyg-9 mis-sense mutation (D69G, GAT to GGT) in or1984ts was the only DNA sequence difference identified by Sanger DNA sequencing of PCR amplified fragments that spanned all exons and introns, with complementation tests to verify gene identity as summarized in Table 1.*

*The complex tac-1(ok3305) deletion/insertion allele (see WormBase) was obtained from the CGC. To determine the phenotype of homozygous ok3305 worms, 170 progeny from a single PCR-confirmed ok3305/+ hermaphrodite brood cultured at 20˚C were singled out onto plates as L4s and kept at 20˚C for 48 hours, at which time their broods were scored for viability. 128 (75.3%) of the progeny produced viable broods; 42 (24.7%) of the progeny produced all dead embryos.* *In a separate brood analysis, 20 progeny from one PCR-confirmed ok3305/+ hermaphrodite were singled onto plates and allowed to produced broods; 5 produced all dead embryos and PCR detected only the deleted allele (ok3305/ok3305); 9 produced viable broods and PCR detected both alleles (ok3305/+); 6 produced viable broods and PCR detected only the WT allele (+/+), also consistent with Mendelian segregation. We conclude that the null phenotype for tac-1(ok3305) is recessive, maternal-effect embryonic lethality.*

*To determine the phenotype of homozygous zyg-9(or1985) worms, 189 progeny from two PCR-confirmed or1985/+ hermaphrodite broods cultured at 20˚C were singled out as L4s and kept at 20˚C for 48 hours. 156 (82.5%) of the progeny produced viable broods, 33 (17.5%) of the progeny produced all dead embryos. In a further test, 20 progeny from one PCR-confirmed or1985/+ hermaphrodite brood cultured at 20˚C were singled out onto plates as L4s and kept at 20˚C for 48 hours, at which time their broods were scored for viability. 6 produced viable broods and PCR detected only the WT band (+/+), 11 produced viable broods and PCR detected both bands (or1985/+), and 3 produced all dead embryos and PCR detected only the mutant band (or1985/or1985). We conclude that the null phenotype for zyg-9(or1985) is recessive, maternal-effect embryonic-lethality, although there may be a delay in larval development, or low penetrance larval lethality, that we missed, given that less than 25% of the progeny from heterozygous parents were homozygous mutant and the singled out L4s were from early in the broods of the two PCR-confirmed or1985/+ parents.*

*We used PCR and Sanger DNA sequencing to verify the presence of the TS tac-1 and zyg-9 mis-sense mutations in all GFP::TBB-2 and GFP::ASPM-1 strains, to ensure that all strains used for identifying tac-1 and zyg-9 requirements were of the correct genotype.*

And from the legend to the new Table 1 summarizing this genetic data:

***Table 1. Genetic analysis of zyg-9 and tac-1 TS and null alleles.*** *Embryonic viability in broods produced by hermaphrodites of indicated genotypes cultured at indicated temperatures. The embryonic lethality observed at 26˚C but not at lower temperatures in broods from ok3305/+ and or1985/+ hermaphrodites is curious and may represent maternal-effect haploinsufficiency but requires further analysis.*

Lastly, we do not include meiotic cell division phenotype data on these null alleles, as that would require extensive effort and is beyond the scope of this manuscript. We will note that the temperature-sensitivity of the deletion heterozygotes is very curious and requires further analysis. We also have observed that the brood sizes show a substantial temperature sensitivity (brood sizes at 26˚C are roughly 1/10 the brood sizes at 15˚C for the homozygous deletion adults). We also imaged *ok3305* and *or1985* mutant embryos at 15˚C and observed strong mitotic defects in the first embryonic mitosis that resemble the strong RNAi knockdown mitotic phenotypes for these loci. We have not included these latter data in this revised manuscript but plan to include them in a subsequent manuscript characterizing the meiotic and mitotic cell division defects in these null mutants.

The time of temperature shift for Fig. 1 needs to be stated in the results text and in the legend given the importance given to rapid shift in the introduction. The text does not state whether the worms were shifted to 26 overnight or if they were shifted 60 seconds before nuclear envelope breakdown.

We apologize for completely failing to describe when we did the temperature upshifts such that the oocytes were at the restrictive temperature throughout meiosis I. The upshifts were done shortly before nuclear envelope breakdown in whole mount worms that were raised and kept at 15˚C by mounting them onto the temperature-controlled stage in a temperature-controlled 15˚C room. This is now described in the text as follows (new text *underlined)*:

*We next assessed how effectively these TS alleles reduce gene function relative to previous studies that have used RNAi to knock down ZYG-9 and TAC-1 function. After TS mutant oocytes were shifted from the permissive to the restrictive temperature shortly before NEB and maintained at the restrictive temperature throughout meiosis I (see Materials and Methods), we compared the defects observed during spindle assembly to the defects previously reported following strong RNAi knockdowns (Matthews et al., 1998; Yang et al., 2003; Chuang et al., 2020).*

And the details are now in the Methods (new text *underlined)*:

***Temperature-shift experiments***

*For imaging control and TS mutant oocytes kept at the permissive or restrictive temperature throughout oocyte meiosis I, worm strains were maintained at the permissive temperature of 15˚C until young adults were mounted on agar pads in a 15˚C cold room and maintained at 15˚C on a metal block until transfer to the CherryTemp chip (see above) pre-set at 15˚C or 26˚C and mounting on the spinning disk microscope. Imaging was begun upon identification of a pre-nuclear envelope breakdown oocyte.*

“We next assessed how effectively these TS alleles reduce gene function.” This is followed by showing phenotypes similar to the Chuang 2020 paper which did not show the extent of knockdown by Western or IF. Thus the phenotypes being similar does not show “how effectively these TS alleles reduce gene function.”

We agree and we have modified the text to say what we intended but admittedly did not say in our original submission, as follows (also shown above):

*We next assessed how effectively these TS alleles reduce gene function relative to previous studies that have used RNAi to knock down ZYG-9 and TAC-1. We compared the defects observed during spindle assembly when TS mutant oocytes were shifted from the permissive to the restrictive temperature shortly before NEB and maintained at the restrictive temperature throughout meiosis I (see Materials and Methods), to the defects previously reported following strong RNAi knockdowns (Matthews et al., 1998; Yang et al., 2003; Chuang et al., 2020).*

The argument that upshifted mutants do not have a prior cage defect (with microtubules passing through the center rather than being restricted to the outside of a cage) is not supported by the cited figures 2B-D and Fig. S3. In almost all of the images (eg. 2A, 2B, 2C), there are microtubule bundles present in the middle of the cage. This is possibly because z-projections are shown. If the authors wish to argue that there are empty cages with no microtubules in the middle, they need to find a different way to display this data, possible by showing single focal planes rather than z-projections. Unless better examples are shown, readers will be suspicious of the quantification in 2I, 2K. The methods state that cages were scored for 3D rotating projections in Imaris but supplemental videos of this are not cited at the relevant location in the text.

We apologize for not better referring to the relevant supplemental movies and we agree the maximum intensity projections of all focal planes are not helpful for documenting that microtubule bundles pass through the interior of the nucleus as the envelope is breaking down. We have now modified Figure 2 to show maximum intensity projections of the three focal planes that encompass the assembling spindle after NEB to better document this defect in TS mutant oocytes. These images admittedly are of low resolution, due to our imaging having to be done within whole mount worms to reproducibly capture these very early stage in spindle assembly, but we do believe they better document this defect in the revised Figure 2. We also now clearly refer in the text to three different supplemental movies (instead of just one in the original submission) that document this defect by virtue of using Imaris software to rotate the corresponding 3-D images from our imaging data sets).

We have added text to the Figure 2 legend to explain these revised images as follows (new text *underlined)*:

*(E-H) during the multipolar stage, in oocytes expressing GFP::TBB-2 and mCherry::H2B. Oocytes depicted in F-H have cage structure defects (for 3-D images of TS mutant oocytes with cage stage defects, see Movies S9, S10 and S11). Note that the cage stage images are maximum intensity projections of 3 focal planes (3 μm) that encompass the dis-assembling nucleus, with limited resolution due to in utero imaging. All other images are maximum intensity projections of all 20 focal plans. For images of cage stage control and mutant oocytes that project all 20 focal planes, see Fig. S5.*

We also changed the relevant Results section heading as follows (new text *underlined)*:

***ZYG-9 and TAC-1 prevent the splitting of early pole foci and may limit their growth during bipolar spindle formation***

We have added text to better refer to the supplemental movies as follows (new text *underlined)*:

*However, when we examined zyg-9 and tac-1 mutant oocytes that failed to establish a bipolar spindle after multipolar stage temperature upshifts, we found that the cage structures had formed properly prior to the upshifts, with microtubule bundles restricted to the periphery (Fig. 2B-D, Fig. S5, see Movie S8 for an example of a mutant oocyte with normal cage structure but defective spindle polarity). These results suggest that the pole coalescence defects in zyg-9 and tac-1 do not depend on an earlier detectably abnormal cage structure.*

And also:

*Importantly, bipolar spindles assembled and separated chromosomes into two equal sets in all seven of the mutant oocytes in which we observed early cage structure defects, indicating that cage structure defects are not sufficient to cause later coalescence defects (Fig. 2J, see Movies S9, S10 and S11 for examples of TS mutant oocytes with abnormal cage structures that nevertheless formed bipolar spindles that separated chromosomes into two sets). Based on these temperature upshift and downshift experiments, we conclude that the pole coalescence defects observed during prometaphase in zyg-9 and tac-1 mutants occur independently of the earlier cage structure defects.*

Table I. **n is quite low** (both for events per embryo and total number of embryos) for making the argument that pole splitting occurs more frequently in upshifted mutants than in controls.

These temperature shifts are challenging experiments, but we now include data from several more temperature shifts at this stage. We have increased the numbers of upshifted *zyg-9(or1984ts)* oocytes from 5 to 9 oocytes, and the number of downshifted *zyg-9(or1984ts)* oocytes from 4 to 10, as shown in the revised table, which is now Table 2, with the results now better supporting our conclusions. We focused on *zyg-9(or1984ts)* because it has the most penetrant spindle defects of the two TS mutants analyzed.

The data in figure 4 showing that the rate of increase in the volume and intensity of GFP::ASPM foci is greater in zyg-9 mutants after upshift is very nice. The opposite effect after downshift is even more dramatic. However, the fact that this difference was not observed in the tac-1 mutant means that this is not related to the significant phenotypes which are the same between zyg-9 and tac-1. Thus the data does not support the conclusion that “To summarize, these changes in pole dynamics after temperature upshifts and downshifts suggest that ZYG-9 and TAC-1 promote pole coalescence both by promoting pole stability and by limiting pole growth.” The sentence explaining this in the discussion is not adequate to deal with this problem.

As noted in response to the more general initial comments above, we thank the reviewer for pointing out this flaw in our logic; we have revised the text as copied above.

The relative lack of multipolar spindles and anaphase defects after late temperature upshifts is used to argue that these phenotypes are due to earlier pole stability defects in early shift experiments. However, it is equally possible that zyg-9 and tac-1 are more completely inactivated by longer temperature shift.

We agree and did not intend to argue that there are not additional requirements after late upshift that we have missed; indeed we emphasized this point in our original submission (see below). Our only intention is to document that there are some requirements that we can detect after our later upshifts. We agree that more complete inactivation might provide more insight. It is possible that the degron tagged allele might prove more useful in this respect, but we believe a detailed phenotype analysis of the new degron-tagged allele we describe (and of the null mutants we describe for the first time) are beyond the scope of this manuscript. We did add text to this section of the manuscript as follows (new text *underlined*):

*While we did not detect any further and possibly more direct roles for this protein complex during chromosome separation after metaphase upshifts, the modestly lower penetrance of the defects observed in TS mutant oocytes maintained at the restrictive temperature throughout meiosis I, compared to those observed after RNAi knockdown (see above), indicates that these TS alleles do not fully reduce gene function at the restrictive temperature. It also is possible that the later upshifts did not allow for enough time to reduce ZYG-9 and TAC-1 function compared to the earlier upshifts. Thus we may not have been able to detect later requirements for lower levels of complex function.*

The spindle rotation defect and polar body extrusion defects are important to analyze but both phenotypes were partial and it is not possible to tell if this is due to partial inactivation. The defects in bipolarity in meiosis II are very weak and do not convincingly show that zyg-9/tac-1 is essential for pole coalescence in meiosis II. It could be that they are essential in meiosis II and the shorter temperature shift is causing less complete inactivation but there is no way to distinguish between these possibilities.

We agree and have modified to the text to emphasize that more complete reduction might result in more severe defects during meiosis II as follows (new text *underlined*).

*We next upshifted control and TS mutant oocytes to the restrictive temperature during meiosis I polar body extrusion, to inactivate ZYG-9 and TAC-1 during meiosis II while bypassing the meiosis I requirements. Spindle pole foci in zyg-9 and tac-1 upshifted oocytes also appeared around the chromosomes but were more dynamic and took longer or failed to assemble into bipolar spindles (Fig. 8F-H, Fig. S10). While the spindle bipolarity and chromosome separation defects in all three mutants were reduced in penetrance during meiosis II compared to meiosis I (Fig. 1I, 1J; Fig. 8I, 8J), these results indicate that ZYG-9 and TAC-1 also influence pole coalescence and spindle bipolarity during meiosis II. As these TS mutations do not appear to reduce gene function as strongly as do RNAi knockdowns, further analysis of the degron-tagged zyg-9 allele might reveal more penetrant and severe defects during meiosis II.*

The data availability statement does not state where the data can be found.

We have now uploaded all original data files, which we had not done previously.

Methods
Somewhere in the paper, the authors should justify the argument that the observed phenotypes are caused by the cited mutations in zyg-9 or tac-1 and are not caused by linked second site mutations. For example, if these mutations were previously complemented with a transgene, this could be stated at the beginning of the Methods. For example after “C. elegans strains used in this study are listed in Table S1.”.

See our response to above comments as to how we have addressed these concerns with alternative genetic approaches, including our isolation and genetic characterization of newly described and likely null alleles for *tac-1* and *zyg-9*. It seems very unlikely that second site mutations would cause the same defects in three independently isolated alleles of two genes. However, this point is perhaps of more concern with respect to changes in the ASPM-1 pole foci growth dynamics we observe in *zyg-9(or1984ts)* oocytes. We now describe our new genetic data, and qualify our conclusions on the pole foci growth dynamics, as described above.

The methods should state how the temperature of the worms was determined. If the authors did not directly measure the temperature of the worms and instead relied on the temperature of the cherry biotech device, at the very least, room temperature should be given. The oil-immersion objective acts as a heat sink and could significantly change the temperature at the worm. This is important for others to be able to reproduce these results.

We have used a temperature probe to verify that the CherryTemp devices we use achieve the correct temperatures in the agar pads. Also, our CherryTemp device includes a feedback system that monitors the temperature of the objective and maintains the objective temperature at the desired setting. We now note this feature of the CherryTemp device in revised Methods text as follows (new text *underlined)*:

*In utero live imaging of oocytes was accomplished by mounting young adult worms with single row of embryos to a 5% agarose pad on a 24x40mm glass coverslip with 1.5 µl each of 15°C M9 buffer and 0.1 µm polystyrene microspheres (Polysciences Inc.), and were gently covered with a 18x18mm coverslip. Samples were then transferred to a fluidic temperature-controlled CherryTemp chip (CherryBiotech) that enabled imaging at precisely the indicated temperature and rapid temperature-shift experiments. We monitor the temperature of agar pads using a probe attached to a thermometer to verify the accuracy of our device, and the CherryTemp device monitors the temperature of the objective and adjusts device flow to accommodate for increased objective temperatures.*

The methods should state the pixel size in the raw images. The cited camera has 16 um pixels so that with no empty magnification, images would have 160 nm pixels but the microscope might have some empty magnification. The methods should also state the approximate laser power used. This information is important for others to reproduce these results.

We have added this information to the Methods as follows (new text *underlined*):

*All imaging was performed using a Leica DMi8 microscope outfitted with a spinning disk con- focal unit–CSU-W1 (Yokogawa) with Borealis (Andor), dual iXon Ultra 897 (Andor) cameras, and a 100x HCX PL APO 1.4–0.70NA oil objective lens (Leica). With the 100x objective, we measured the pixel size to be 0.1470 +/- 0.0005 μm, versus the theoretical pixel size of 0.160 μm from the camera’s 16 x 16 μm pixels, indicating a small amount of empty magnification within the system. Metamorph (Molecular Devices) imaging software was used for controlling image acquisition. The 488nm and 561nm channels were imaged simultaneously every 10s with 1μm Z-spacing for a total stack size of 20μm.*

**Reviewer 2 comments.** Assembly of the acentrosomal spindle in C. elegans is not entirely understood. One big limitation to our understanding the roles played by specific proteins is the lack of tools to acutely deplete or inactivate them. In the current manuscript, Harvey & Bowerman address the role of the ZYG-9/TAC-1 complex during different steps of meiosis I from spindle assembly to anaphase chromosome segregation using fast-acting temperature-sensitive (ts) alleles. These proteins have mainly been studied using RNAi-mediated depletion (with the exception below). These alleles allowed the authors to dissect the requirements of these proteins with high temporal resolution.

The main findings of this manuscript are that 1) ZYG-9 and TAC-1 are required for pole coalescence into a bipolar structure by stabilising pole foci and limiting their growth rate and 2) ZYG-9 and TAC-1 maintain bipolarity during Metaphase I by suppressing ectopic poles, a process important for maintaining chromosome congression. Interestingly, their results are complementary to those posted in a very recent preprint that uses a degron-based strategy for acute ZYG-9 depletion (Mullen et al 2022). This highlights the value of complementary strategies to address meiotic events with high temporal resolution to gain a better understanding of how an oocyte assembles the spindle and how it is dynamically regulated.

Overall, the experiments are well performed and explained in detail, and the results are very clear (including the strength of each phenotype).

We appreciate these positive comments.

• It would be good for the reader to have a schematic of the different mutations within ZYG-9 and TAC-1. This is a central point, as these alleles are the key tool of the paper and having a better idea of whether the alleles operate at the protein level and/or function would hopefully help the reader better understand the penetrance of different phenotypes. Are the proteins still capable of interacting in the different mutants? I can see this was partially addressed for some mutants and their impact during mitosis in Bellanger et al, 2007.

We agree it is helpful to include a schematic and now do so as a new supplemental figure. We could not easily fit the schematic as a panel into Figure 1, and decided it made more sense to have it as a supplemental figure. We also indicate the nature of the deletion alleles in this new supplemental figure. We do not know if these mutations prevent the proteins from interacting during meiosis and consider such an analysis beyond the scope of this study.

• I also think it would be good for the reader to have a schematic of the temperature shift in relation to meiosis progression to add a visual cue for better orientation in the figure. While t=0 is explained in the methods section, it could be relevant to include these in the main text and/or Fig.

As noted in comments to Reviewer #1, we failed to clearly indicate when we did our initial upshifts for Figure 1 and now include a more detailed description of that procedure in both the text and the methods, as noted above. We also include a detailed supplemental figure (Figure S2) that was also in our original submission but we now cite it earlier and throughout the text whenever referring to temperature shift experiments. This figure provides all information as to when the different upshift and downshifts during the multipolar and bipolar stages were done. Given the somewhat different meiosis progression times in the different mutants, and the more rapid initial progression through meiosis I in downshifted oocytes relative to upshifted oocytes (see Figure S2), it is not possible to easily make a schematic that suffices for all three mutants, but Figure S2 provides complete detail on all upshifts and downshifts. As noted, we now cite it earlier in the text before describing any of the experimental outcomes, as follows (new text *underlined*):

*To observe oocyte meiotic spindle assembly dynamics in control and TS mutant oocytes, we have used spinning disk confocal microscopy, coupled with a microfluidics temperature-control system, to image live oocytes in utero within immobilized whole-mount worms from transgenic strains that express both a GFP-tagged ß-tubulin (GFP::TBB-2) to mark microtubules, and an mCherry-tagged histone (mCherry::H2B) to mark chromosomes (see Materials and Methods). To confirm the presence of the TS mutations, we isolated genomic DNA from each TS mutant strain used for imaging and used DNA sequencing to confirm the presence of the intended mutant alleles (see Materials and Methods). All live imaging was done using oocytes within whole mount worms, which limits resolution but was essential for accurately timing when temperature shifts were done relative to NEB, the earliest step in oocyte meiotic spindle assembly (see Fig. S2).*

• Figures could benefit from having arrows pointing to relevant characteristics mentioned in the text.

We have added additional arrowheads and asterisks to Figures 2, 3 and 6 to better indicate examples of extra or split poles and also three-way anaphase outcomes, with chromosomes separated into 3 sets.

• Are the levels of the proteins altered in the mutants? Since these proteins form a complex, analysis of their levels in the different mutants could be interesting. Either WB of endogenous proteins or maybe using fluorescently labelled ts alleles.

We have not addressed whether these mutations affect the protein levels. While this might be interesting, we do not think it is important for defining gene requirements: whether the proteins are stable or unstable, and present or absent, does not affect our conclusions as to the nature of the requirements. While it might help to understand how the different mutations result in the defects (by being absent versus present but non-functional), we believe such analysis is beyond the scope of this manuscript and does not affect our conclusions as to the nature of the gene requirements and phenotypic defects we have identified. We do think our addition of new genetic data, including a characterization of likely null alleles for both *tac-1* and *zyg-9*, is significant and further confirms that the defects we describe are due to loss of function of these genes, as noted above in response to Reviewer 1 comments.

• For Figure 3, the text states that since spindle assembly and chromosome segregation were often defective in all three ts mutants at 26 deg, elevation of MT levels cannot account for the bipolarity and segregation defects. what is the incidence of defective bipolarity within the movies with normal MT levels?

As we note in the discussion, we do not think that an overall elevation in microtubule levels precludes localized excessive microtubule polymerization more being responsible for the spindle defects we describe. Indeed, as XMAP215/ZYG-9 orthologs have been shown in vitro to have both microtubule polymerase and de-polymerase activity, it is likely that both activities are important for its functions, possibly even in the same context and process. Also, the levels of microtubules vary substantially over time in any one oocyte, and it is not necessarily meaningful to single out oocytes with low levels during some intervals but not others. We therefore have not assessed if low average microtubule levels correlate with less severe defects, but given the lack of correlation among the different mutant alleles, we think this is unlikely. However, we have modified the text to more clearly state the lack of correlation we note as follows (revised text *underlined*):

*Surprisingly, spindle microtubules levels were significantly increased only in tac-1(or455ts) oocytes but not in zyg-9(or623ts) or zyg-9(or634ts) oocytes, relative to control oocytes maintained at 26°C (Fig. 3A, 3B). Because spindle assembly and chromosome separation were often defective in all three TS mutants when maintained at 26˚C, and because the penetrance of the spindle defects were highest in zyg-9(or1984ts) and lower in tac-1(or455ts)* oocytes*, a simple elevation in overall spindle microtubule levels alone cannot account for the spindle bipolarity and chromosome separation defects observed after reducing ZYG-9 or TAC-1 function (see Discussion).*

• In Figure 5, is the resolution seems particularly low and it does not help to easily identify ectopic microtubule bundles.

As we now describe in the text in more detail, the resolution in our live imaging experiments is relatively low due to all of the imaging being done in whole mount worms, which significantly lowers resolution compared to isolated oocytes. However, to capture the earliest stages in spindle assembly and properly time temperature upshifts and downshifts, we can only use whole mount worms for these experiments. Hence the resolution is limited, but it is sufficient to see a strong correlation between the appearance of ectopic GFP::ASPM-1 foci or abnormal microtubule bundles, and the displacement of bivalents away from the metaphase plate. We acknowledge the limitations of resolution in new text at the beginning of our revised Results section as follows:

*All live imaging was done using whole mount worms, which limits resolution but was essential in order to accurately time when temperature shifts were done relative to NEB, the earliest step in oocyte meiotic spindle assembly (see Fig. S2).*

Have all data underlying the figures and results presented in the manuscript been provided?
Large-scale datasets should be made available via a public repository as described in the *PLOS Genetics* [data availability policy](https://urldefense.com/v3/__http:/journals.plos.org/plosgenetics/s/data-availability__;!!C5qS4YX3!GqRQMbWv9Co4O9hx61aB-RbF2-3Om2TluhdLv7dS6TtI6GBWwzg6cXCb8m5kSw3Drd6HWVNAHKQWBTQXbWo$), and numerical data that underlies graphs or summary statistics should be provided in spreadsheet form as supporting information.

Reviewer #1: No: Means and SEM are provided in figures but I do not see a supplemental spreadsheet with all of the raw vales. The journals policy on the website says that "available upon request" is not adequate.

Reviewer #2: Yes

We have provided spreadsheets with all data sets used to calculate means and SEMs, as now indicated in the Materials and Methods as follow (new text *underlined*):

*P-values comparing distributions for all scatter plots were calculated using the Mann–Whitney U-test. P-values comparing slopes were calculated using a two-tailed t-test. Statistical analysis was performed using Microsoft Excel (Microsoft) and Prism 9 (GraphPad Software) and graphs were made in Prism 9. Raw data for all statistical analyses are provided in Excel spreadsheets (see Supplemental Information).*
